# Association between circulating levels of heat-shock protein 27 and aggressive periodontitis

**DOI:** 10.1007/s12192-018-0891-4

**Published:** 2018-05-15

**Authors:** Frank Kaiser, Nikos Donos, Brian Henderson, Rajesh Alagarswamy, George Pelekos, David Boniface, Luigi Nibali

**Affiliations:** 1 Department of Microbial Diseases, Eastman Dental Institute, University College London, London, UK; 2 Centre for Immunobiology and Regenerative Medicine and Centre for Oral Clinical Research, Institute of Dentistry, Barts and the London School of Medicine and Dentistry, Queen Mary University London, Turner Street E1 2AD, London, UK; 3 Faculty of Dentistry, The University of Hong Kong, Pokfulam, Hong Kong; 4 Biostatistics Unit, Eastman Dental Institute, University College London, London, UK

**Keywords:** Periodontitis, Stress proteins, Hsp27, Inflammation, Cytokines

## Abstract

Heat-shock protein (Hsp) 27 is a major intracellular molecular chaperone and controller of intracellular responses to inflammatory signals. In the extracellular space, recombinant Hsp27 has been described to exert anti-inflammatory activities*.* The aim of this study was to assess the association between circulating levels of Hsp27 and different types of periodontitis. Pro- and anti-inflammatory cytokines and the stress proteins Hsp27 and Hsp60 with proposed anti- and pro-inflammatory properties, respectively, were measured by two-site ELISA in the serum of patients with aggressive periodontitis (AgP, *n* = 30), chronic periodontitis (CP, *n* = 29) and periodontally healthy controls (H, *n* = 28). Furthermore, Hsp27 and Hsp60 levels were also measured longitudinally in 12 AgP patients at 6 time points up to 3 months after treatment. AgP patients had lower levels of Hsp27 compared to CP patients and healthy subjects (adjusted one-way ANOVA, *p* < 0.001, followed by post hoc Tukey HSD comparisons), while no differences in levels of Hsp60 or cytokines between the three groups were detected. In CP patients and H subjects, the systemic Hsp27 levels correlated with Hsp60 (*r* = 0.43, *p* < 0.001; *r* = 0.59, *p* < 0.001, respectively) and with pro-inflammatory cytokines TNF-α (*r* = 0.48, *p* < 0.001; *r* = 0.55, *p* < 0.001, respectively) and IL-6 (*r* = 0.44, *p* < 0.01). However, no such correlations were detected in AgP cases. No consistent temporal patterns of changes of Hsp27 concentration were detected across AgP patients following periodontal treatment. This study provides the first evidence that Hsp27 may be differentially expressed and regulated in AgP patients as compared with CP patients and healthy individuals.

## Introduction

The periodontal diseases are the world’s most common chronic inflammatory diseases (Eke et al. 2012) and are currently subdivided into a number of diagnostic categories (Armitage 2004). Of these, the two principal forms of periodontitis, chronic (CP) and aggressive periodontitis (AgP), have been suggested to differ in clinical appearance and disease progression (Armitage and Cullinan 2010). However, objective clinical measures have thus far failed to discriminate between these two perceived conditions (Frydman and Simonian 2011). For example, these gingival diseases cannot be histologically (Smith et al. 2010) or microbiologically (Armitage 2010) discriminated, and analysis of gene expression profiles (Kebschull et al. 2013) or measures of systemic inflammation (Cairo et al. 2010) have not proved diagnostically discriminatory.

There is a very large number of proposed diagnostic and prognostic biomarkers, although very few of them have bridged the translational gap in inflammatory diseases (Mischak et al. 2012). One of the most intriguing groups of potential biomarkers are cell stress proteins. These include various families of protein-folding proteins known as molecular chaperones and protein-folding catalysts whose intracellular levels have mostly been reported to increase in stressed cells (Saibil 2013). However, several studies have observed a decrease in intracellular Hsp70 and Hsp90 levels in monocytes and polymorphonuclear cells, along with a pattern of early extracellular induction in acute conditions like sepsis (Vardas et al. 2014; Papadopoulos et al. 2017; Spanaki et al. 2018). It is rapidly becoming established that these proteins are pivotal controllers of cellular proteostasis, the network of competing and integrated biological pathways that controls protein biogenesis, folding, trafficking and degradation. Defective proteostasis is associated with cell and tissue pathology (Jovaisaite et al. 2014; Wang et al. 2014). It has been recognised since the 1970s that cell stress proteins can be secreted by cells and can enter into the circulation (Henderson and Pockley 2005). In more recent years, cell stress proteins have been proposed as useful biomarkers (Henderson and Pockley 2012). For example, Hsp72 levels were able to discriminate sepsis from systemic inflammatory response syndrome (SIRS) and furthermore, they were strong predictors of mortality (Vardas et al. 2017). Unlike some other biomarkers, cell stress proteins exert potent intercellular signalling activities with properties resembling both pro- or anti-inflammatory cytokines (Henderson and Pockley 2010; Pockley and Henderson 2018).

In a previous trial, our group assayed plasma obtained from patients with chronic periodontitis and control subjects for systemic levels of a number of cell stress proteins, Hsp10, Hsp60 and BiP (Henderson and Pockley 2010). Levels of Hsp10 and BiP were significantly lower in the blood of patients compared with controls, while Hsp60 levels were similar in both groups. Effective treatment of periodontitis was associated with a significant increase in Hsp10 levels to those found in controls and a lesser increase in BiP levels with no change in Hsp60. This initial evidence shows the potential importance of circulating cell stress proteins in periodontitis and its response to treatment.

In this study, we concentrated on another cell stress protein, the myeloid cell-modulating cell stress protein Hsp27, which has been described to exert anti-inflammatory activities (De et al. 2000; Laudanski et al. 2007). The main outcome was Hsp27 serum concentration and the null hypothesis was that systemic Hsp27 and Hsp60 levels do not vary in AgP and CP, as compared to healthy subjects. Furthermore, we explored associations between Hsp27 and Hsp60 and key inflammatory and anti-inflammatory cytokines as well as longitudinal changes in Hsp27 following periodontal treatment in AgP.

## Methods

### Subject selection, clinical evaluation and periodontal treatment

The analyses described here consisted of a case-control and a treatment study. Both studies were reviewed and approved by the UCL/UCLH joint ethics committee (references 05/Q0502/84 and 07/H0713/74), and suitable individuals referred for care at the Eastman Dental Hospital gave written informed consent to take part. All participants were of Caucasian origin. Exclusion criteria for both studies were presence of other chronic oral conditions as assessed by the examining clinician: pregnancy; reported history of medical conditions or concomitant systemic medications; intake of anti-inflammatory drugs or antibiotics within 3 months; known infectious diseases and previous periodontal therapy within 12 months. At the baseline visit, the patients’ height, weight and waist circumference were taken and BMI calculated. A single calibrated examiner measured full-mouth probing pocket depth (PPD), clinical attachment loss (CAL) and full-mouth bleeding on probing score (FMBS) at six sites per tooth as previously described (Guerrero et al. 2005). Repeated measurements of 10 non-study subjects (with at least 15 min separation) showed 98% intra-examiner repeatability within 2 mm for PPD.

The case-control study consisted of a total of 87 consecutive ethnically Caucasian subjects drawn from a larger case-control study, based on the following characteristics:

- Periodontally healthy controls (H, *n* = 28) with PPD ≤ 4 mm and FMBS <20%
- Chronic periodontitis (CP, *n* = 29), with at least 10 sites with PPD > 4 mm
- Aggressive periodontitis (AgP, either localised or generalised, *n* = 30), with PPD and CAL ≥ 6 mm and bleeding on probing in at least three sites in different teeth

Periodontitis patients were diagnosed with AgP based on self-report of familial aggregation and systemic health and on rapid progression evidenced by either age at diagnosis < 45 years old or radiographic evidence of bone loss > 1 mm in at least two non-adjacent sites within a 1-year period (Mombelli et al. 2002).

The treatment study included 12 AgP patients (diagnosed as above) less than 45 years old. All treatment sessions were performed by a single qualified periodontist as described before (Nibali et al. 2013). Briefly, after a baseline visit, full-mouth non-surgical periodontal therapy (NSPT) was performed under local anaesthesia on day 14, and follow-up oral hygiene instructions were provided. Patients were reassessed an average of 30 days after NSPT. On day 70, periodontal surgical treatment (open-flap debridement, OFD) was performed under local anaesthesia in the worst affected quadrant (residual > 5 mm PPDs with bleeding on probing). Full-thickness muco-periosteal flaps were elevated, granulation tissue and subgingival debridement and, if required, localised non-supporting bone (osteoplasty) removed, and the flaps were readapted and sutured. Patients were seen for follow-ups and blood samples taken on day 1, 7, 30, 60 and 90 after OFD surgery.

### Blood sampling

Blood samples were collected at the baseline visit for both investigations. For the intervention study, blood samples were additionally collected at all follow-up appointments. All patients had been fasting for 6 h prior to blood sampling on all visits. Blood was collected by venipuncture from the antecubital vein and serum was immediately separated by centrifugation. Serum samples were kept frozen at − 80 °C until the analyses were performed.

### Hsp27 measurement

**Fig. 1:**
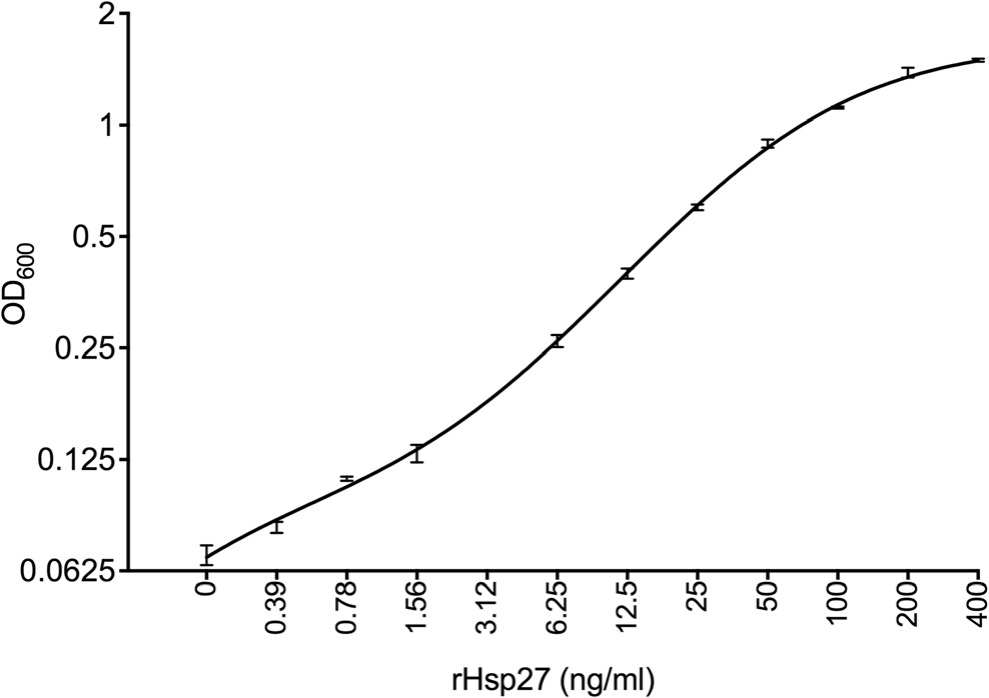
Evaluation of the Hsp27 immunoassay (ELISA). The graph, a representative standard curve of the Hsp27 ELISA assay, shows the measurement of serial dilutions (1:2 in PBS) of recombinant Hsp27 starting at a concentration of 400 ng/ml. Input rHsp27 concentrations (ng/ml) are plotted versus optical density (OD_600_) after assay development, with both axes on log_2_ scale. Error bars show the standard deviation of triplicate sample measurements

Hsp27 in serum was measured using an in-house-developed two-site enzyme-linked immunosorbent assay (ELISA). Briefly, 96-well plates (Immuno MaxiSorp, Nunc) were coated with a mouse monoclonal anti-HSP27 antibody (clone 2A5, ATGen) at 1 μg/ml in coating buffer (50 mM carbonate-bicarbonate buffer, pH 9.6) overnight at 4 °C (see Fig. 1). Plates were washed with wash buffer (PBS, 0.05% Tween-20), and non-specific binding sites were blocked by incubation with 2% (*w*/*v*) bovine serum albumin (BSA Chromatopur Immunoassay Grade, MP Biomedicals) in PBS for 1 h at room temperature. After washing, human recombinant Hsp27 protein (ATgen; 0–200 ng/ml) or dilutions (PBS) of serum samples and controls were added and incubated for 2 h at room temperature. Plates were washed extensively with wash buffer and incubated for 2 h at room temperature with a goat polyclonal anti-Hsp27 antibody (C-20, Santa Cruz; 1 μg/ml in 0.5% BSA/PBS). After washing, bound goat anti-hsp27 antibody was detected by incubation with HRP-conjugated donkey polyclonal anti-goat IgG (Santa Cruz ssc-2304, mouse and human adsorbed, 40 ng/ml in 0.5% BSA in wash buffer) at room temperature for 45 min. Binding of conjugated antibody was detected using TMB substrate (affymetrix/eBioscience), and the reaction was stopped by adding 2 N H_2_SO_4_. Absorbance at 450 nm, using a 570-nm reference wavelength, was determined with a plate reader (MRX II, Dynex). Cytokine concentrations were calculated by the plate reader software (Revelation, Dynex). Each serum sample was assayed in triplicate. The assay was highly specific for Hsp27 and showed no cross-reactivity with recombinant human Hsp10, Hsp60, or Hsp70 stress proteins and did not cross-react with bovine serum albumin, foetal calf serum or *E. coli* lipopolysaccharide (data not shown). The detection limit of the assay was 0.1 ng/ml Hsp27, and the linear range was between 1 and 50 ng/ml Hsp27 (Fig. 3). The intra- and inter-coefficients of variability were less than 7%. The assay showed almost linear dilution of serum samples with PBS. The recovery rate of exogenously added recombinant Hsp27 to serum from donors with low (1 ng/ml) or medium levels (15 ng/ml) of endogenous Hsp27 was ≥ 95%.

### Hsp60 measurement

Hsp60 was measured by ELISA as previously described (Shamaei-Tousi et al. 2006) with few modifications. Hsp60 in serum or recombinant Hsp60 protein (ATgen; 0–5 μg/ml) was captured with a mouse monoclonal anti-Hsp60 antibody (clone LK1, Stressgen; 1 μg/ml). A goat polyclonal anti-Hsp60 antibody (Stressgen; 1 μg/ml) was used as detection antibody, followed by incubation with a biotinylated donkey polyclonal anti-goat IgG (Alpha Diagnostics, 1:5000). Bound secondary antibody was detected with Streptavidin-HRP (Biolegend; 0.25 μg/ml). The assay was developed with TMB substrate (affymetrix/eBioscience), and the reaction stopped with 2 N H_2_SO_4_.

### Cytokine measurements

Serum levels of IL-1β, IL-6, IL-10 and TNF-α were measured by two-site ELISA using commercial kits according to the manufacturer’s recommendations (Human Ready-SET-Go!® ELISA, affymetrix/eBioscience).

### Statistical analysis

Since no data on Hsp27 relative to periodontal disease are available in the literature, no formal sample size calculation was performed. All data were analysed using SPSS Statistics software package (V24, IBM). Differences in the characteristics between the case-control study groups were computed with parametric (independent-samples *t* test) or non-parametric (Mann-Whitney U Test) tests dependent upon the data frequency distribution and equality and homogeneity of variance tests. Categorical variables were analysed by chi-square testing. Because the distribution of heat-shock protein and cytokine concentrations in blood was markedly skewed, data was natural log-transformed (LN) (after adding a constant) to achieve a more normal distribution prior to statistical analysis. Differences in analyte concentrations between subject groups were analysed by parametric independent-samples *t* tests or one-way between-group (ANOVA) tests followed by a post hoc comparison using the Tukey HSD test. Parametric correlation analysis (Pearson) was performed to evaluate the association between cytokine and heat-shock protein concentrations in serum. Age, gender, BMI and cigarette smoking were included in all models as covariates. A two-sided value of *p* < 0.05 was considered statistically significant.

## Results

### Case-control-study

**Fig. 2:**
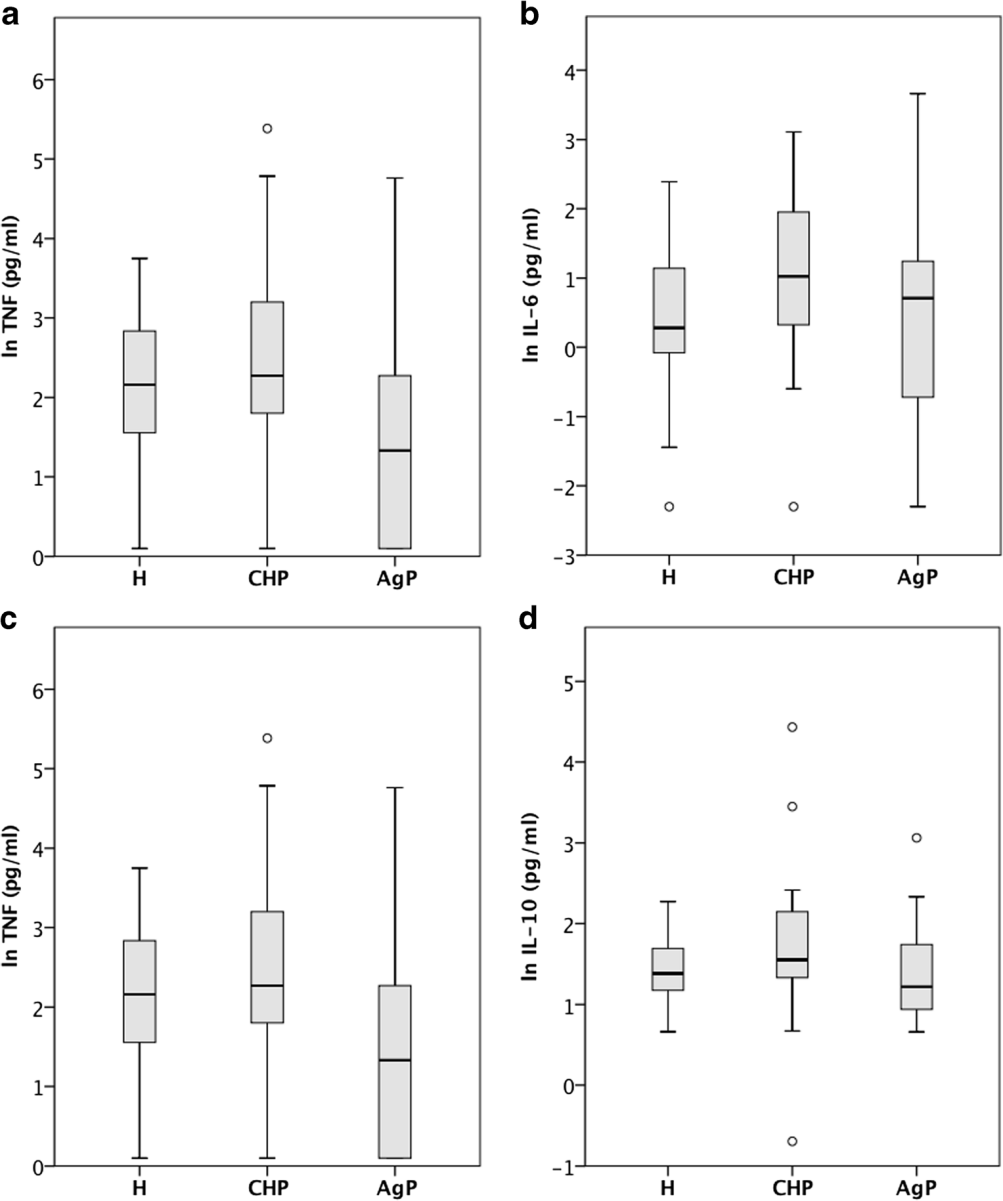
**a**–**d** Pro- and anti-inflammatory cytokine concentrations in the circulation were similar between healthy subjects and patients with different forms of periodontitis. Cytokine concentrations in serum of healthy subjects (H, *n* = 28) and patients diagnosed with periodontitis classified into aggressive periodontitis (AgP, *n* = 29) and chronic periodontitis (ChP, *n* = 29). The concentrations of IL-1 (**a**), IL-6 (**b**), TNF-α (**c**) and IL-10 (**d**) were measured by two-site ELISA. The data distribution of transformed cytokine concentration is visualised as box-and-whisker plots (SPSS). The boxes represent the interquartile range and the line inside the box refers to the median value. The whiskers extend to the smallest and largest values within 1.5 times the interquartile range; outliers (as defined by SPSS) are visualised as open circles

The main characteristics of AgP and CP patients and healthy controls are reported in Table 1. Baseline concentrations of systemic levels of the pro-inflammatory cytokines IL-1, IL-6 and TNF-α and the anti-inflammatory cytokine IL-10 were compared between healthy subjects and patients diagnosed with AgP or CP (Fig. 2). There was no statistically significant difference in the cytokine levels between the three groups for IL-1 (*p* = 0.078; Kruskal-Wallis test), IL-6 (*p* = 0.39; ANOVA) and IL-10 (*p* = 0.93; ANOVA). TNF-α levels were reduced in AgP patients (*p* = 0.02; ANOVA).

**Table 1:** Comparison of demographic characteristics, clinical parameters and serum baseline measurements of patients and control subjects

	AgP	CP	H	*p* value
Cases [*n*]	30	29	28	
Age [years]	35.5 (5.0)	42.2 (8.2)	39.4 (11.2)	0.011
Male gender [%]	30	37.9	32.1	0.801
Smokers current [%]	46.7	37.9	10.7	0.01
BMI	26.4 (7.0)	25.4 (6.0)	22.8 (2.2)	0.036
Sites with PPD > 4 mm [*n*]	67.5 (39.2)	44.1 (24.9)	0	< 0.001
IL-1 [pg/ml]	7.9 (107.8)	13.4 (247.3)	16.9 (122.1)	0.078
IL-6 [pg/ml]	1.9 (39.0)	2.7 (22.4)	1.2 (10.8)	0.039
TNF [pg/ml]	2.4 (104.6)	7.8 (192.2)	6.9 (37.4)	0.02
IL-10 [pg/ml]	2.9 (19.5)	4.2 (83.7)	3.5 (7.8)	0.093
Hsp60 [ng/ml]	51.3 (9088)	136.7 (10,000.0)	90.3 (3023.3)	0.172
Hsp27 [ng/ml]	1.7 (25.0)	4.7 (37.6)	4.9 (3.5)	< 0.001

The table shows the characteristics of subjects included in both studies. Continuous demographic and clinical variables (age, BMI and PPD) are reported as mean and standard deviation, serum analytes (IL-1, IL-6, TNF, IL-10, Hsp60, Hsp27) as median and interquartile range. The last column reports the statistical significance (*p* value) of the differences between the three groups. Categorical variables were analysed by chi-square test for independence. The differences for continuous variables were computed with parametric (one-way between-groups ANOVA) or non-parametric (Kruskal-Wallis) tests dependent upon the data frequency distribution. Serum analyte data were transformed as described in the “Material and methods” section prior to statistical analysis

*AgP* aggressive periodontitis, *CP* chronic periodontitis, *H* healthy subjects, *BMI* body mass index, *PPD* pocket probing depth

**Fig. 3:**
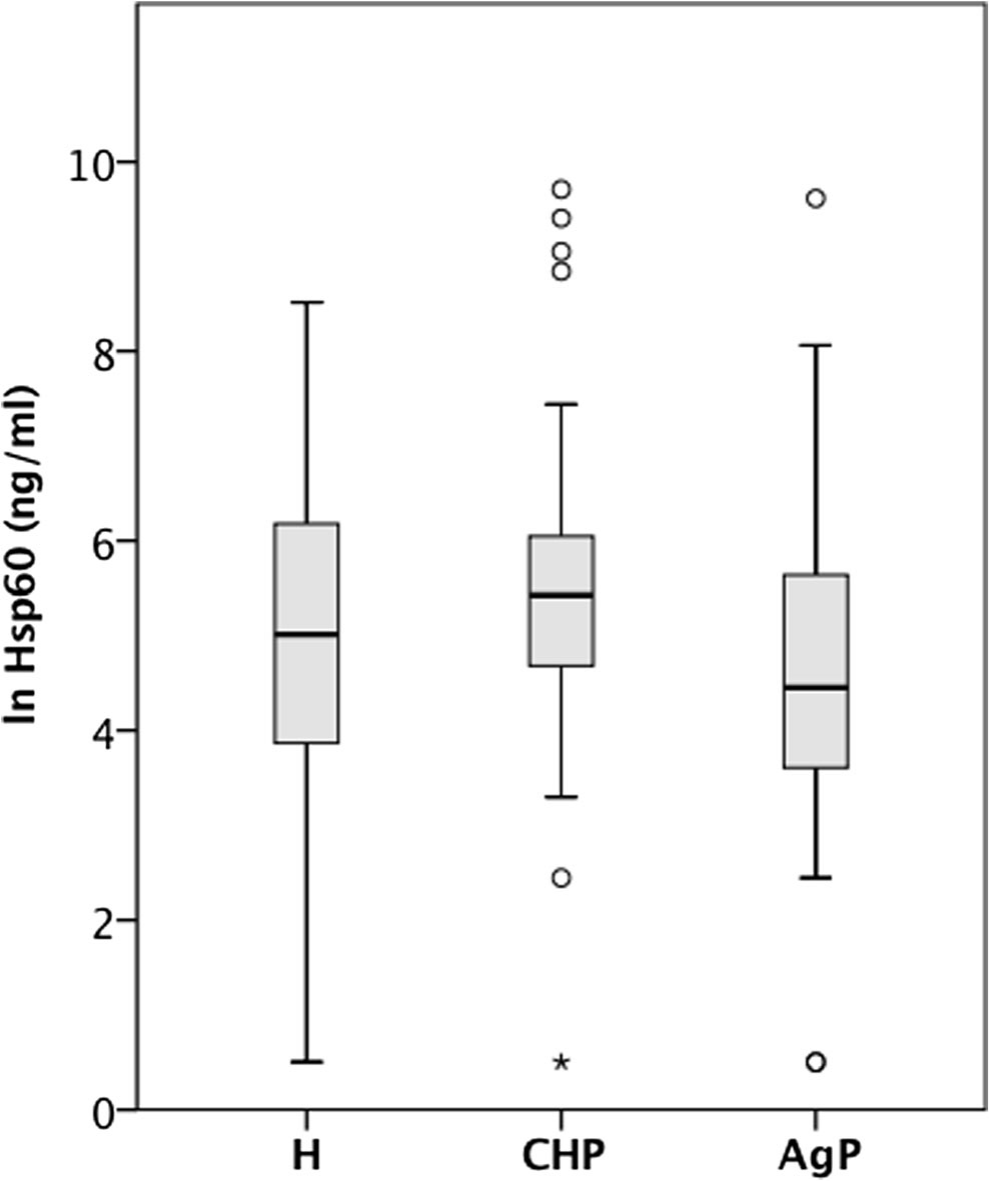
Systemic levels of Hsp60 were similar in healthy individuals and patients with different forms of periodontitis. The concentration of Hsp60 in the serum was measured by two-site ELISA. The distribution of transformed Hsp60 concentrations in the serum of healthy subjects (H, *n* = 28) and patients diagnosed with chronic (ChP, *n* = 29) or aggressive (AgP, *n* = 30) periodontitis is visualised as box-and-whisker plots (SPSS)

**Fig. 4:**
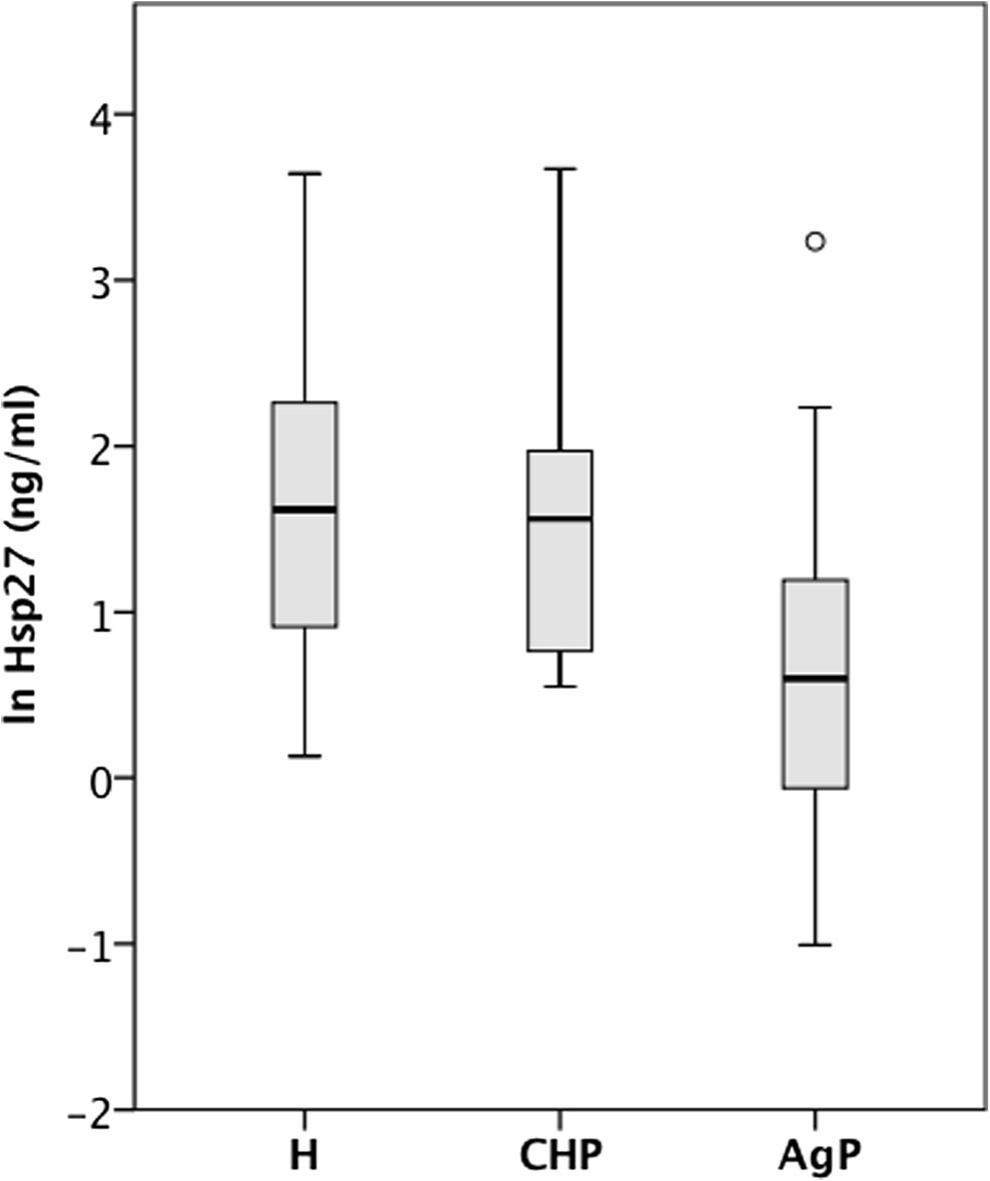
Patients diagnosed with aggressive periodontitis had significantly lower levels of Hsp27 in their circulation. The concentration of Hsp27 in the serum of healthy donors (*n* = 28), patients with chronic (*n* = 29) or aggressive (*n* = 30) periodontitis, was measured by two-site ELISA. The box-and-whisker plot shows the distribution of Hsp27 concentrations after transformation

Hsp60 serum concentrations were not significantly different (*p* = 0.172, one-way ANOVA) between healthy individuals and patients with periodontitis (Fig. 3). The analysis of Hsp27 concentrations in the serum of H, AgP or CP patients revealed a statistically significant difference between the three subject groups (one-way ANOVA, *p* < 0.001) (Fig. 4). Post hoc tests (Tukey HSD) indicated that patients diagnosed with AgP had significantly less Hsp27 (M = 0.62, SD = 0.97) in their circulation than healthy subjects (M = 1.68, SD = 0.97) or CP patients (M = 1.59, SD = 0.84). We further analysed the effect of possible known confounding factors (age, gender, BMI, smoking, and PPD > 4 mm) on Hsp27 levels. Standard multiple regression analysis identified smoking as a confounding factor for Hsp27 serum levels (*p* = 0.004). However, after adjustment for smoking, age, gender, BMI and number of PPD, the detected association between AgP and Hsp27 levels remained statistically significant (ANCOVA, *p* = 0.013).

The relationship between serum concentrations of systemic stress proteins (Hsp27, Hsp60) and cytokines (IL-1, IL-6, TNF-α, IL-10) in healthy individuals and CP or AgP patients (Table 2) was investigated using Pearson correlation coefficient analyses. Preliminary analyses were performed to ensure no violation of the assumptions of normality, linearity and homoscedasticity. We observed complex patterns of positive associations which markedly differed across the subject groups. In both AgP and CP but not H subjects, strong associations between IL-1 and IL-10 or TNF-α (*r* > 0.52, *p* < 0.01), as well as between IL-10 and TNF-α (*r* > 0.47, *p* < 0.01), were detected. The association patterns of Hsp60 were similar between the three subject groups: Hsp60 showed a strong positive association with both IL-6 and TNF-α (*r* > 0.53, *p* < 0.01), but only weak associations with IL-1 and IL-10 in all groups. In sharp contrast, Hsp27 levels were strongly associated with Hsp60 and TNF-α (*r* > 0.43, *p* < 0.01) in healthy individuals and CP patients, whereas no such associations could be revealed in AgP patients.

**Table 2:** Associations between circulating heat-shock proteins Hsp27 and Hsp60 and cytokines in healthy controls and patients with periodontitis

	Hsp27	Hsp60	IL-1	IL-6	IL-10
a. H					
Hsp60	0.599**				
IL-1	0.059	0.177			
IL-6	0.353	0.381*	− 0.132		
IL-10	0.09	− 0.118	0.058	0.562**	
TNF	0.553**	0.753**	0.326	0.321	0.007
*n*	28	28	28	27	27
b. CP					
Hsp60	0.439*				
IL-1	0.254	0.377*			
IL-6	0.446*	0.538**	0.346		
IL-10	0.357	0.374*	0.525**	0.453*	
TNF	0.482**	0.672**	0.545**	0.453*	0.479**
*n*	29	29	29	29	29
c. AgP					
Hsp60	0.33				
IL-1	0.084	0.343			
IL-6	− 0.031	0.455*	0.637**		
IL-10	0.064	0.313	0.632**	0.620**	
TNF	0.193	0.557**	0.576**	0.688**	0.698**
*n*	29	29	29	29	29

The tables show the *R* values of the parametric correlation analysis (Pearson) for serum levels of Hsp27, Hsp60 and cytokines IL-1, IL-6, IL-10 and TNF in (a) healthy subjects, (b) patients diagnosed with chronic periodontitis and (c) patients with aggressive periodontitis

Correlation is significant at the **p < 0.01 level (two-tailed); *p < 0.05 level (two-tailed)

### Treatment study

**Fig. 5:**
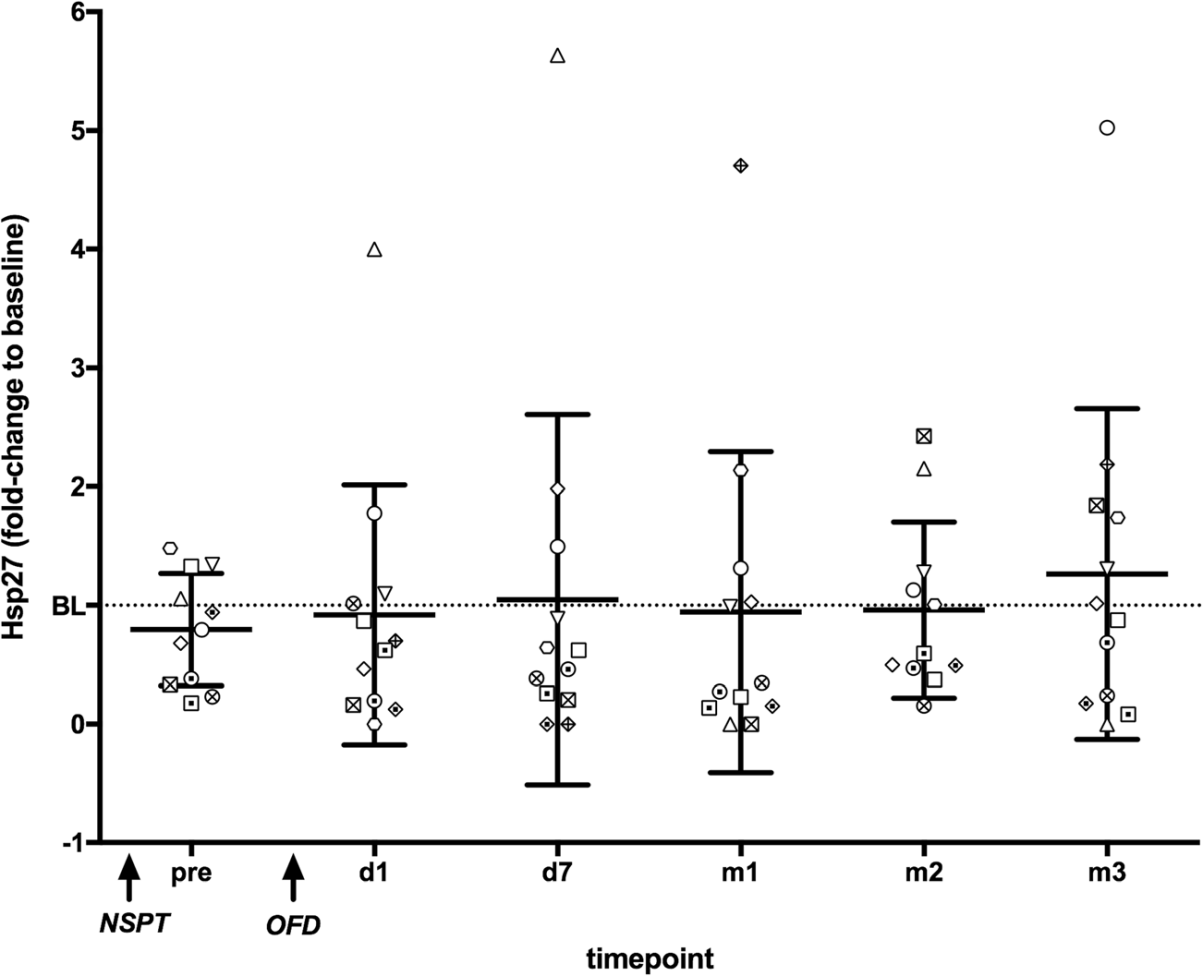
Periodontal treatment in patients with aggressive periodontitis induces complex and dynamic changes of Hsp27 concentration in their circulation. The graph shows Hsp27 concentrations in the serum of 12 AgP patients who have undergone periodontal treatment. Hsp27 concentrations (fold-change) are shown over time as compared to baseline (BL) Hsp27 levels determined in serum samples taken prior to periodontal treatment. Hsp27 was measured 6 weeks (pre) after non-surgical periodontal treatment (NSPT) and 1 and 7 days (d) and 1, 2 and 3 months (m) after periodontal surgery (OFD, open-flap debridement performed 2 months post-NSPT)

Hsp60 and Hsp27 serum levels were measured in patients with AgP (*n* = 12) at various time points after induction of periodontal therapy (as described in the “Material and methods” section). The concentration of Hsp60 in the serum of these patients remained largely unchanged upon periodontal treatment (data not shown). In sharp contrast, these patients showed profound and highly dynamic changes in circulating Hsp27 levels after non-surgical (NSPT) and surgical (OFD) treatment (Fig. 5). However, the temporal patterns resulting from fluctuating Hsp27 levels after treatment were highly heterogeneous across AgP patients and no consistent direction or magnitude of Hsp27 response could be observed in the treatment time points investigated.

## Discussion

This study is the first to suggest that systemic concentrations of Hsp27 may differ between different types of periodontal disease. Periodontitis is characterised by the development of an exacerbated host inflammatory response leading to destruction of tooth-supporting hard and soft tissue and, ultimately, to tooth loss. Its prevalence is high and currently increasing in industrialised populations, with a recently increased prevalence of severe periodontitis up to nearly 10% (Eke et al. 2012). Based on recent surveys, approximately half of the US and half of the UK population suffer from periodontitis (Eke et al. 2012). Periodontitis is heterogeneous and currently classified into two main forms: chronic periodontitis (CP) and the rarer aggressive periodontitis (AgP) which is estimated to affect approximately 1–2% of individuals (Albandar and Tinoco 2002). AgP usually affects younger systemically healthy individuals and has a more rapid rate of progression. However, there is currently no objective biomarker-based diagnostic tool or sufficiently distinct histopathological characteristics to discriminate the two forms of periodontitis (Armitage and Cullinan 2010), so the current classification is under revision.

Molecular chaperones, also referred to as “cell stress proteins” or “heat shock proteins” (HSPs)—as their levels often increase during cell stress (such as elevated temperature), include a repertoire of functions such as *de novo* protein folding, disaggregation and protein trafficking. Initially, cell stress proteins were assumed to be purely intracellular proteins (Wick et al. 2004; Henderson and Pockley 2005). However, it has been demonstrated that in addition to their intracellular chaperoning functions, cell stress proteins can be released by cells and exist on the outer plasma membrane or in extracellular environments such as body fluids (Panayi et al. 2004). These secreted versions of cell stress proteins are found in the circulation in health and in a range of diseases and are increasingly being used as biomarkers in human disease states. Currently, we have most information about the role of circulating cell stress proteins in the cardiovascular diseases (Shamaei-Tousi et al. 2007b; Henderson and Pockley 2012) and in acute conditions like sepsis (Vardas et al. 2014, Briassouli et al. 2014). The secreted versions of stress proteins are hypothesised to have a broad spectrum of biological activities, depending on the particular protein and its concentration. Numerous studies have demonstrated that cell stress proteins have potent intercellular signalling actions for example with leukocytes and vascular endothelial cells, with activity profiles similar to those of pro- and anti-inflammatory cytokines (also referred to as “chaperokine” function) (Henderson and Pockley 2010). This suggests that cell stress proteins may function, like cytokines, as extracellular homeostatic control proteins or as homeostatic controllers of immunity (Henderson 2010; Henderson and Kaiser 2013). For example, the secreted pro-inflammatory cell stress protein cyclophilin A has been proposed as a therapeutic target in atherosclerosis (Bukrinsky et al. 2013) as well as a diagnostic maker for vascular disease in type 2 diabetes (Ramachandran and Kartha 2012). The heat-shock protein Hsp10 and the endoplasmic reticulum molecular chaperone BiP (HSPA8) have been demonstrated to have significant anti-inflammatory actions (Vanags et al. 2006; Panayi and Corrigall 2014) and both are currently undergoing clinical investigation. However, the role of other stress proteins such as Hsp72 is still less clear, with human studies showing either a protective role or an association with mortality and infections in sepsis (Briassoulis et al. 2014; Levada et al. 2018).

With regard to the role of secreted cell stress proteins in periodontitis, both bacterial and host heat-shock proteins are hypothesised to contribute to pathology. To date, only a few studies have investigated the potential role of circulating levels of human cell stress proteins in the periodontal disease pathogenesis (Nethravathy et al. 2014; Tsybikov et al. 2014). Notably, our group has demonstrated that circulating levels of Hsp10 and of BiP, both considered anti-inflammatory agents, were significantly higher in healthy controls than in patients with periodontitis, suggesting that these two cell stress proteins were either not being secreted or were being removed from the circulation in patients with periodontitis (Shamaei-Tousi et al. 2007a).

Hsp27 is a major intracellular molecular chaperone and controller of intracellular responses to inflammatory signals (Alford et al. 2007). Recombinant Hsp27 is also able to modulate myeloid cell activity with the protein showing an anti-inflammatory mode of action in vitro (De et al. 2000)*.* Furthermore, Hsp27 in the circulation has been demonstrated to function as an atheroprotective protein by preventing the release of inflammatory cytokines (Rayner et al. 2009; Józefowicz-Okonkwo et al. 2009)*.* Hsp27 is also expressed in numerous human cancers, and its expression seems to correlate with poor clinical outcome (Ciocca and Calderwood 2005). In particular, treatments aimed at modulating Hsp27 levels and particularly its effects on apoptosis are being experimented on for prostate cancer treatment (Yu et al. 2017). The role of systemic Hsp27 in periodontitis is currently unknown and we are not aware of any studies investigating it. Interestingly, the serum concentrations of key cytokines, typically involved in the induction and resolution of inflammatory processes, failed to distinguish between disease status or between the two forms of the disease, AgP and CP, respectively. Notably, we found that the concentration of Hsp27 in peripheral blood in AgP patients was significantly lower than in CP patients or in healthy controls. This finding suggests that in AgP patients, either less Hsp27 is being secreted or Hsp27 is being removed from the circulation at a greater than normal rate (Lebherz-Eichinger et al. 2012). In line with the described promotion of anti-inflammatory signalling by released Hsp27 (De et al. 2000; Rayner et al. 2008), the identified lower levels of this stress protein in the blood of AgP patients may result in impaired immune-dampening activity thereby contributing to an exacerbation of periodontal disease.

Our (Nibali et al. 2013) and other groups (Becerik et al. 2012; Mattuella et al. 2013) have previously investigated to which extent local and systemic levels of cytokines may contribute to the manifestation of severe periodontal disease. We have also proposed the hypothesis that cell stress proteins and cytokines generate complex intra- and extracellular networks, which function in the control of cells to external and internal stressors (Henderson and Kaiser 2013; Kaiser et al. 2014). The existence of such networks would suggest that the cell stress response is a key parameter in cytokine network generation and, as a consequence, in control of immunity. This raises the question whether the two heat-shock proteins investigated in this study, Hsp27 and Hsp60, may be part of a cell stress protein-cytokine network controlling inflammation in periodontal disease. Interestingly, when we analysed the association between circulating levels of Hsp27 and concentrations of key cytokines involved in the onset of inflammation (IL-1β, IL-6, TNF-α) as well as resolution of inflammation (IL-10), we detected distinct differences in the HSP-cytokine associations between healthy subjects, CP and AgP patients. While Hsp27 correlated positively with Hsp60, TNF-α and to a lesser extent IL-6 in both healthy subjects and CP patients, there were no associations for Hsp27 detectable in the AgP subgroup. In sharp contrast to the Hsp27-cytokine associations, Hsp60 concentrations correlated positively with IL-6 and IL-10 levels in all groups. These results support the hypothesis that highly complex HSP-cytokine networks control the inflammatory processes in periodontal diseases and emphasise that the composition or function of such networks may be different in clinically distinct forms of periodontitis.

Treatment of AgP patients resulted in significant changes in circulating hsp27 levels in all individuals. Interestingly, the temporal Hsp27 response patterns were highly dynamic, showing that its levels both increased and decreased by different magnitude after individual treatments. However, no clear pattern of change was observed across all treated cases. As no untreated controls were available for comparison, it is not clear whether the observed changes were due to periodontal treatment or were part of normal fluctuations of Hsp27 systemic concentrations.

The lack of associations between Hsp27 and cytokines suggests that this particular heat-shock protein may prospectively be investigated as a novel, possibly cytokine-independent, molecular regulator for the resolution of inflammation in periodontitis. One hypothesis is that stress proteins are interacting with pro-inflammatory target cells in an attempt to dampen their activity and control inflammation and, in essence, are being used up. However, it should be noted that the trauma and release of a huge burden of microbes from the gingival pockets upon treatment also has the potential to influence directly the expression and release of Hsp27 into the circulation. Further studies in large cohorts need to investigate systemic levels of heat-shock proteins after periodontal treatment (Shamaei-Tousi et al. 2007a).

Limitations of this study include absence of untreated controls in the treatment part, absence of investigation of local (oral or subgingival) Hsp27 levels and a relatively limited sample size. The strengths lie in the analysis of a range of periodontal phenotypes (healthy, AgP, CP) and a range of cytokines and heat-shock proteins, including the novelty of Hsp27 analysis. Our finding that the concentration of Hsp27 in the serum of patients with periodontitis significantly differs between distinct forms of the disease suggests that this protein may be differently regulated in different forms of periodontitis. This finding should be investigated in independent larger populations, should be extended to other HSPs and should include analysis of local/intracellular HSP levels, before the possible pathogenic effect of Hsp27 and its potential to serve as a biomarker for clinical purposes are considered.
